# Multivisceral resection of nonfunctional pancreatic neuroendocrine neoplasm with nearby organ invasion: a case report

**DOI:** 10.3389/fendo.2023.1236685

**Published:** 2023-09-26

**Authors:** Cong Zhang, Weiqiao Niu, Yaopeng Xu, Yijie Lu, Lining Huang, Song Li, Xinwei Jiang, Jianwu Wu

**Affiliations:** Department of Hepatobiliary Surgery, The Affiliated Suzhou Hospital of Nanjing Medical University, Gusu School, Nanjing Medical University, Suzhou, China

**Keywords:** pancreatic neuroendocrine neoplasm, multivisceral resection, invasion, liver metastasis, debulking surgery

## Abstract

Pancreatic neuroendocrine neoplasms (pNENs) are relatively rare epithelial malignancies originating from pancreatic neuroendocrine cells, pathologically classified into well-differentiated pancreatic neuroendocrine tumors (pNETs) and poorly-differentiated pancreatic neuroendocrine carcinoma (pNECs). Although they also fall under the category of pNENs, the almost entirely distinct biological characteristics and survival prognosis have caused debate among surgeons when it comes to the development of surgical intervention options, particularly for locally advanced G3 pNETs and pNECs. We present a case of 66-year-old male with nonfunctional G3 pNET, invasion of five nearby pancreatic organs and type II liver metastases. The patient achieved good outcomes after undergoing multivisceral resection and postoperative adjuvant chemotherapy. This finding helps surgeons better understand locally advanced pNENs, formulate treatment decisions systematically and confidently, and balance patient benefits and risks of surgery.

## Introduction

Pancreatic neuroendocrine neoplasms (pNENs) are relatively rare epithelial malignancies originating from pancreatic neuroendocrine cells, and their incidence and frequency have steadily risen over the decades (1). pNENs typically develop insidiously with highly heterogeneous biological behaviors. Based on mitotic count and the Ki-67 proliferation index, pNENs are classified as well-differentiated G1, G2, and G3 pancreatic neuroendocrine tumors (pNETs) or poorly-differentiated pancreatic neuroendocrine carcinomas (pNECs) (2). They may undergo transformation as the disease progresses, manifesting as inert growth, aggressive growth, or even early metastasis. Therefore, there are various hurdles and unresolved issues in clinical disease diagnosis and therapy.

Surgery is the major strategy for achieving a good long-term prognosis for pNENs. They can be classified into resectable, borderline resectable, and locally advanced tumors according to the stage and surgical possibility (3, 4). Among them, borderline resectable and distant metastatic tumors often increase the difficulty of surgery due to invasion of nearby organs or a significant tumor load, including the expansion of the scope of surgery and the inability to achieve radical resection (5). It is worth noting that there is ongoing debate among surgeons on the survival and symptomatic benefits of surgery for patients with locally advanced and metastatic pNENs, particularly G3 pNETs and pNECs (6). The survival advantages and symptom improvement brought by surgery may be superior to systemic comprehensive treatment, according to increasing amounts of evidence (7–11). This unquestionably serves as a compelling reason for doctors to determine whether to have surgery, even while the procedure is still scheduled.

We herein report a valuable case in which a patient with nonfunctional G3 pNET experienced local invasion of five abdominal organs and liver metastasis. The surgical procedure and postoperative adjuvant therapy enhanced the patient’s long-term survival rate and quality of life.

## Case description

A 66-year-old male patient was hospitalized in April 2022, complaining of a massive retroperitoneal tumor that was revealed by health screen half a month ago. A hypoechoic lesion measuring up to 110 mm by 60 mm was seen on the initial ultrasound scan in the region of the left kidney and the spleen.

**Figure 1:**
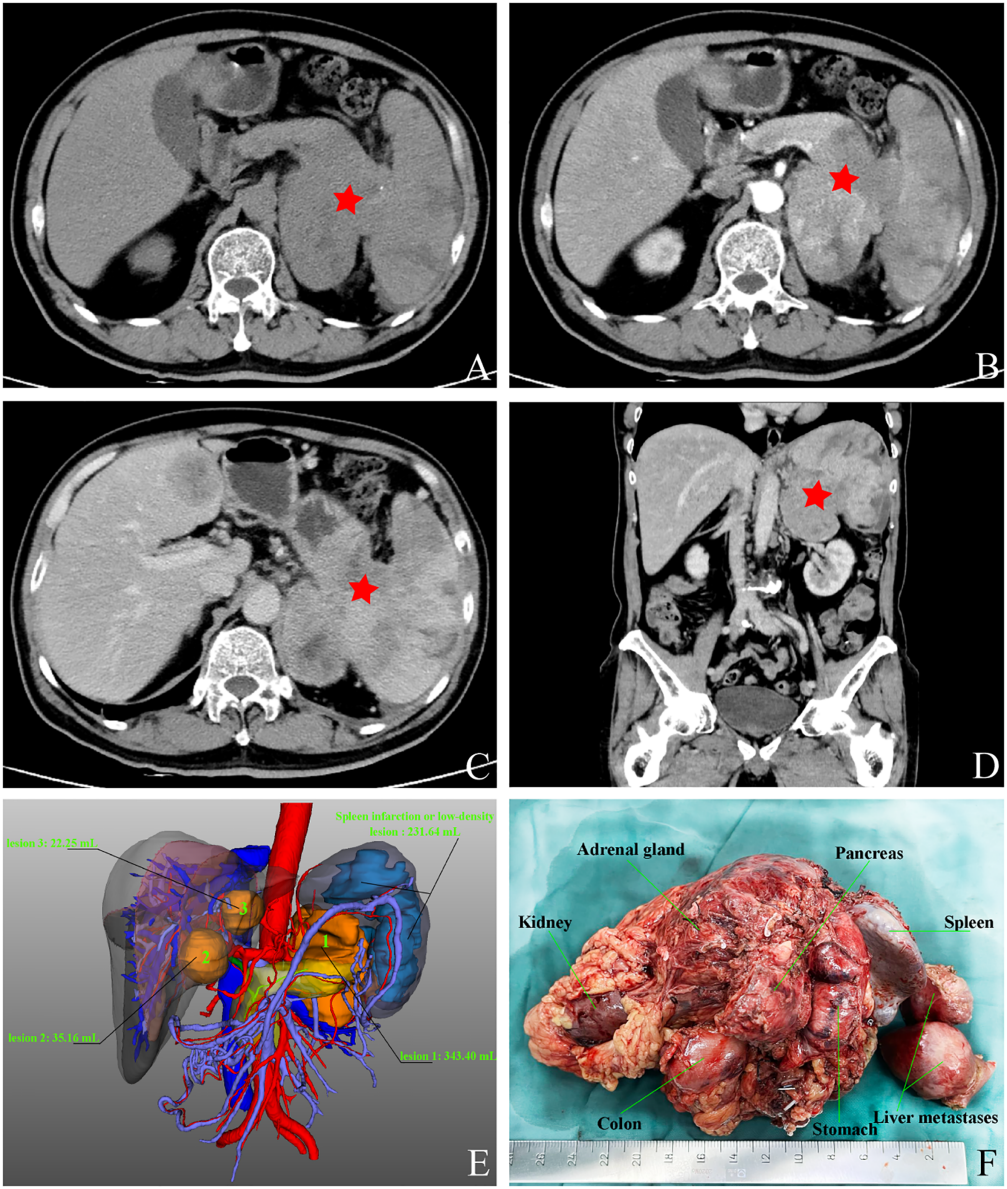
Images of abdominal computed tomography and 3D visualization reconstruction, macroscopic specimen after multivisceral resection. **(A)** plain scan; **(B)** arterial phase; **(C)** venous phase; **(D)** coronary scan; **(E)** visualization reconstruction; **(F)** postoperative specimen.

After admission, the patient underwent a comprehensive and systematic examination. The patient’s physical examination showed no obvious positive signs, and his overall condition was acceptable according to the preoperative laboratory results (**Table 1**). Importantly, no obvious abnormalities were found in common tumor markers, and hormone levels used for differential diagnosis of functional endocrine tumors were within normal limits. The patient underwent an abdominal contrast-enhanced computed tomography (CT) scan, as follows (**Figures 1A-D**): there was a 8.1×6.0×7.9 cm solid mass of the left adrenal gland involving the pancreatic tail, serosal surface of the gastric fundus, and spleen with splenic infarction. The tumor thrombi invaded the splenic artery, resulting in stenosis of the arterial lumen, and tumor thrombi were also seen in the splenic vein. Two metastatic tumors could be seen in the left lobe of the liver. CT image data stored in the form of DICOM was imported into Yorktal 3D+Visualization System for 3D visualization reconstruction (**Figure 1E**).

**Table 1:** The preoperative laboratory data.

Hematology						Coagulation		
WBC	3.68×10^9^	/L	ALP	76	U/L	PT	12.9	Sec↑
RBC	3.92×10^12^	/L↓	*γ*-GTP	29	U/L	APTT	28.8	sec
Hb	106	g/L↓	CHE	267	U/L			
PLT	168×10^9^	/L	BUN	6.45	mmol/L			
			Cre	74.0	μmol/L			
Biochemistry			Na	144.7	mmol/L	Serology		
TP	68.1	g/L	K	4.27	mmol/L	CRP	3.64	mg/L↑
Alb	39.36	g/L	Cl	109.7	mmol/L			
TBIL	8.5	μmol/L	Ca	2.17	mmol/L	Tumor marker		
DBIL	2.7	μmol/L	FPG	5.57	mmol/L	CEA	1.73	ng/mL
GPT	9	U/L	TG	2.81	mmol/L	AFP	2.80	ng/mL
GOT	11	U/L↓	TC	2.89	mmol/L	CA19-9	12.30	U/mL
LDH	128	U/L				CYFRA21_1	2.15	ng/mL↑

WBC, white blood cell; RBC, red blood cell; Hb, hemoglobin; PLT, platelet count; TP, total protein; Alb, albumin; TBIL, total bilirubin; DBIL, direct bilirubin; GPT, glutamic pyruvic transaminase; GOT, glutamic oxaloacetic transaminase; LDH, lactate dehydrogenase; ALP, alkaline phosphatase; γ-GTP, gamma glutamyl transpeptidase; CHE, cholinesterase; BUN, blood urea nitrogen; Cre, creatinine; FPG, fasting plasma glucose; TG, triglyceride; TC, total cholesterol; PT, prothrombin time; APTT, activated partial thromboplastin time; CRP, C-reactive protein; CEA, carcinoembryonic antigen; AFP, Alpha-fetoprotein; CA19-9, carbohydrate antigen 19-9; CYFRA21_1, Cytokeratin 19 fragment. “↑, ↓” represent respectively an increase or decrease in examination index compared to the normal range.

**Figure 2:**
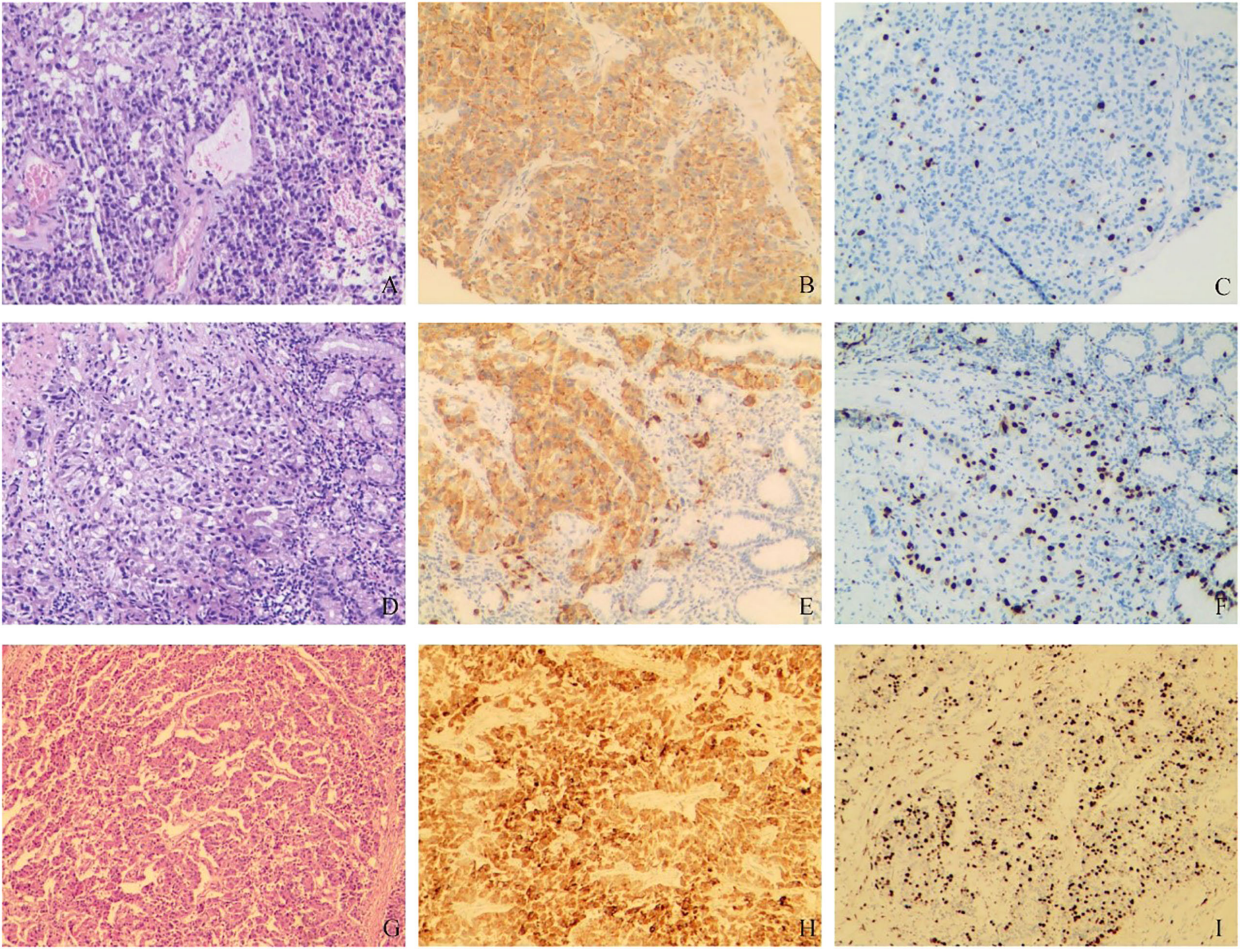
Pathological findings. **(A–C)** HE and immunohistochemical staining of tumor puncture tissue in the left adrenal region: **(A)** HE staining suggested tumor cell showed a nest-like distribution, neoplastic cells with polygonal shape, abundant and acidophilic cytoplasm, mile nuclear atypia without necrosis, rare nucleoli; **(B)**. CgA (+); **(C)**. Ki-67 (10%). **(D–F)** HE and immunohistochemical staining of gastric fundus tumor tissue: **(D)** HE staining showed the microscopic characteristics of tumor cells were similar to A; **(E)** CgA (+); **(F)** Ki-67 (20%). **(G–I)** HE and immunohistochemical staining of postoperative tumor tissue: **(G)** HE staining suggested tumor cell showed nest-like and cord-like distributions, neoplastic cells with polygonal shape, abundant and acidophilic cytoplasm, mild-to-moderate nuclear atypia with massive necrosis, obvious nucleoli; **(H)**. CgA (+); **(I)** Ki-67 (40%).

**Figure 3:**
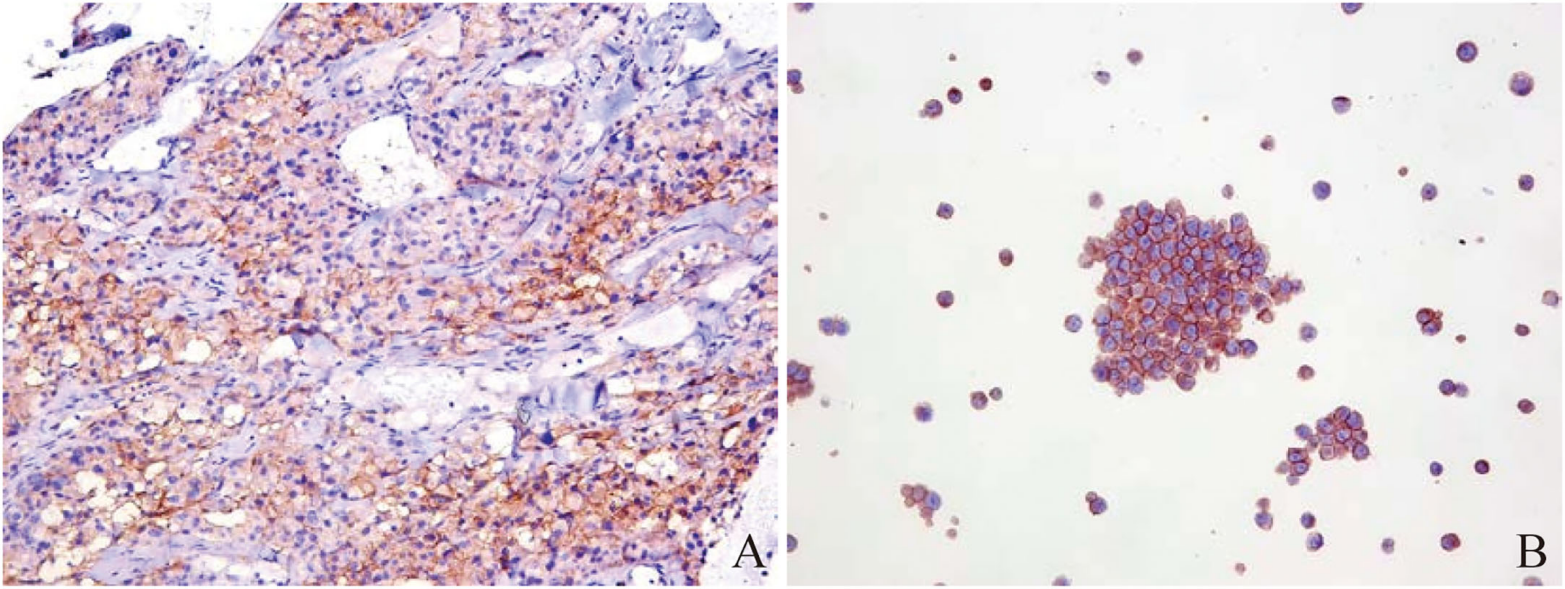
Immunohistochemistry of PD-L1 protein in puncture pathological tissues from Beijing ACCB Biotech Ltd. **(A)** microscopic image of puncture pathological tissue: TPS=20%, CPS=23; **(B)** positive control: TPS ≥1%.

In order to further diagnose the nature and origin of the tumor, the patient underwent an ultrasound-guided puncture biopsy of the tumor tissue in the left adrenal gland and a biopsy of gastric fundus tissue under a gastroscope. Hematoxylin and eosin (HE) staining of two specimens both suggested tumor cells showed a nest-like distribution, neoplastic cells with polygonal shape, abundant and acidophilic cytoplasm, mile nuclear atypia without necrosis, and rare nucleoli (**Figures 2A, D**). Immunohistochemistry staining revealed that the tumor cells were positive for chromogranin A (CgA) (**Figures 2B, E**), synaptophysin (Syn), cytokeratin 18 (CK18) and negative for inhibin, CEA, calretinin, epithelial membrane antigen (EMR), vimentin, Melan-A, Pax-8, SF-1, P53, S-100. The Ki-67 index was 10% in the specimen of the adrenal lesion (**Figure 2C**) and 20% in the specimen of the gastric fundus (**Figure 2F**). Moreover, detection of 168 oncogenes and programmed cell death ligand 1 (PD-L1) protein expression was performed on puncture tissue specimens by Beijing ACCB Biotech Ltd to regulate the use of molecularly targeted drugs, immune checkpoint inhibitors, and chemotherapy drugs and to create precise and effective treatment plans for patients. Detection of 168 oncogenes revealed the inactivation of the *TSC2* gene (c.1793A > G, p.Y598C, mutation abundance=68.69%) and the activation of the *FGFR3* gene (gene amplification, mutation abundance=6.35 Copies) (**Table 2**). Chemotherapy sensitivity testing suggested in the paper that capecitabine had good patient efficacy while etoposide and fluorouracil had good safety. For *TSC2* gene mutations, this paper further recommended the use of the FDA-approved tumor-targeting drugs everolimus and temsirolimus, as well as ponatinib for *FGFR3*, while the other three mutant genes had not yet received approved drugs. Besides, the detection of anti-vascular targets for *FGFR3* suggested that enrotinib, lenvatinib, and pazopanib had further benefits. Detection of the PD-L1 protein expression showed that the tumor proportion score (TPS) was 20% and the combined positive score (CPS) was 23 (**Figure 3**). This implied that PD-L1 immunohistochemistry test was positive, and immune checkpoint inhibitor drugs may be highly beneficial for patients.

**Table 2:** Detection of 168 oncogenes from Beijing ACCB Biotech Ltd.

	Gene	Exon/Base/Amino Acid	Mutation abundance	Sensitive related drugs	Tolerance related drugs	Clinical trials
Tumor targeting/ drug resistance related gene mutations	TSC2	exon17 c.1793A>G p.Y598C	68.69%	none	none	Everolimus (NCT01470209, NCT02352844) Temsirolimus (NCT01596140, NCT02215720)
	FGFR3	gene amplification	6.35 Copies	none	none	Ponatinib (NCT02272998)
Gene mutations without targeted therapy	FGF4	exon2 c.437A>T p.Y146F	57.26%	none	none	none
	FH	exon9 c.1273G>A p.D425N	78.66%	none	none	none
	NBN	exon12 c.1912T>C p.S638p	71.04%	none	none	none

Based on the above examinations, we diagnosed the tumor as a G3 nonfunctional neuroendocrine tumor with multiple organ invasions and metastases, most of which originated in the digestive system. Although the tumor was huge, invading the stomach, spleen, and left adrenal gland, and accompanied by two metastatic lesions on the liver, it did not invade important celiac arteries (celiac trunk artery, superior mesenteric artery, common hepatic artery) and veins (superior mesenteric vein, portal vein) from the CT and 3D visualization reconstruction image. Therefore, either R0 or R1 tumor resection or effective tumor reduction surgery can alleviate the clinical symptoms of patients and may improve their long-term prognosis. The operation was performed by an experienced hepatobiliary surgeon. During the operation, exploration found that, besides the preoperative imaging findings, the tumor also invaded the local colon. At the same time, because the tumor was located behind the adrenal gland and renal artery and vein, which were difficult to expose, the left kidney was removed. Finally, the patient received retroperitoneal tumor resection, partial gastrectomy, pancreatectomy, splenectomy, left nephrectomy, partial colon resection, and enucleation of liver metastases (**Figure 1F**). The pathological diagnosis of surgical specimens was as follows: HE staining suggested tumor cell showed nest-like and cord-like distributions, neoplastic cells with polygonal shape, abundant and acidophilic cytoplasm, mild-to-moderate nuclear atypia with massive necrosis, obvious nucleoli (**Figure 2G**). No tumor involvement was found at the cutting edges of stomach, colon, pancreas, ureter, liver, and no lymph node metastases were found around the tumor (0/10) under the microscope. Immunohistochemistry staining showed that the tumor cells were positive for CgA (**Figure 2H**), Syn, CK18 and negative for P53, Melan-A, inhibin, S-100, Heppar-1, vimentin, TTF1, CT, Pax-8, CDX2. The Ki-67 index was 40% in the specimen (**Figure 2I**). So far, the final diagnosis of this case has been G3 nonfunctional NET, most of which originated in the pancreas, TNM stage: T_4_N_0_M_1a_ (3). The patient has been receiving chemotherapy since May 28, 2022, without recurrence to date. He was treated with 750 mg/m^2^ of capecitabine twice daily from day 1 to 14 and 200 mg/m^2^ of temozolomide once daily from day 10 to 14, repeating such a procedure twice in a cycle of 28 days. A dose reduction or schedule adjustment was performed in consideration of the individual patient’s general condition and toxicity at the discretion of the clinician. Response to treatment was assessed every three months through CT and/or MRI images according to the Chinese guidelines for the diagnosis and treatment of pancreatic neuroendocrine neoplasms (2020) (12), and the patient has been followed up to date without recurrence.

## Discussion

pNENs usually display characteristic organoid growth patterns with typical neuroendocrine morphology. Patients may not have unique symptoms brought on by the tumor mass impact at different stages of the disease. Most of them incidentally discover tumors when getting their health checked. Some individuals may live for many years even with liver metastases, but others may experience a more precipitous clinical course with widespread metastasis and dismal survival. The WHO Classification of Tumors of the Digestive System, 5^th^ edition, modified the classification of neuroendocrine tumors in light of advances in tumor molecular pathology. The new classification eliminates confusion between well-differentiated G3 pNETs and poorly-differentiated pNECs, which are thought to be two separate entities with unique clinical, morphological, and molecular characteristics (13). Patients with G3 pNETs had a significantly longer median overall survival (41–99 vs. 17 months) compared with patients with pNECs (14, 15). Meanwhile, the median overall survival of G3 pNETs after surgery without metastasis (39.2 months), with metastasis (19.5 months) was significantly longer than that of pNECs (respectively 16 months and 9.1 months). Contrarily, overall survival did not significantly differ between surgical and non-surgical procedures among patients with pNECs (16). Therefore, different from the dismal survival prognosis and poor treatment of pNECs (17), the disease treatment and management of G3 pNETs should adopt a more upbeat outlook and more radical surgical intervention.

Accurately classifying G3 pNETs and pNECs has become difficult for surgeons due to variations in clinical presentations and treatment approaches. Although some blood biomarkers, such as CgA, NSE, et al., have specificity for pNETs and pNECs, their sensitivity is constrained and may be influenced by tumor type, tumor burden, and secretion level (18). In addition to conventional CT and MRI, somatostatin receptor imaging, which has a sensitivity of 91% and a specificity of 94% (19), is thought to be the most accurate tool for identifying pNENs and their metastases. ^68^Ga-DOTA-SSA PET/CT is also important for determining radionuclide uptake, which is associated with the response to peptide receptor radionuclide therapy (PRRT) (20). Research has found that ^68^Ga-DOTA-SSA PET/CT can effectively discriminate between low-grade and high-grade pNENs but cannot further differentiate G3 pNETs and pNECs (21). Due to outstanding diagnostic sensitivity of the ^18^F-FDG PET/CT for G3 pNET and pNEC, it is frequently employed in clinical practice in conjunction with 68Ga-DOTA-SSA PET/CT to increase disease diagnostic sensitivity and precise staging (22, 23). These examinations are not commonly accessible, just due to a lack of PET-CT equipment or inadequate testing levels in medical facilities, as well as the expensive examination charges. This has also led to significant regrets and deficiencies in our preoperative examination of this case. Endoscopic ultrasound (EUS) is the gold standard for the diagnosis of pNEN. EUS-guided fine needle aspiration (FNA) for cytology and histology can distinguish G3 pNETs and pNECs, enabling decisions on an appropriate treatment strategy (24). However, when the differential diagnosis of G3 pNETs and pNECs in morphology and immunohistochemistry is difficult, personalized gene detection techniques can describe genetic molecular characteristics and mutated genes, providing a reference for precise treatment of patients while making a differential diagnosis (25, 26). We conducted genetic testing on puncture specimens and revealed genetic mutations. Based on the latest guidelines and research status, we screened drugs that can benefit patients.

The surgical value of borderline resectable or locally advanced G3 pNETs and pNECs is hotly debated. The guidelines of European Neuroendocrine Tumor Society (ENETS) recommend extended multivisceral pancreatic resection with or without combined vascular reconstruction for patients with G1 pNETs and G2 pNETs without distant metastatic disease, but is prohibited for higher-grade G3 pNETs and pNECs (4). National Comprehensive Cancer Network guidelines (27) provide a more detailed overview of G3 pNETs and pNECs based on the biological characteristics (e.g., Ki-67 index, PET imaging) and clinical symptoms of tumors (e.g., tumor burden and complications). Locally advanced or metastatic G3 pNETs with favorable biology can still remove primary and metastatic sites, but pNECs tend to prefer systemic, comprehensive treatment. The Chinese Guidelines for the Diagnosis and Treatment of Pancreatic Neuroendocrine Neoplasms (2020) think that for G3 pNETs and pNECs patients who are not expected to achieve R0 and R1 resection, effective tumor reduction surgery can alleviate the patient’s clinical symptoms and may improve their long-term prognosis. Simple primary lesion resection may also prolong the postoperative survival time of patients with metastatic sites (12). In this case, the patient’s preoperative examination revealed a G3 pNET with type II liver metastases and no evidence of tumor invasion into significant abdominal arteries and veins. Chinese guidelines support concurrent or phased surgical therapy for patients with type II liver metastases when the expected total tumor reduction is higher than 70% (often greater than 90%, including both the metastatic and original lesions). Removing the main lesion while reducing the tumor load on the metastatic lesion as much as feasible is the best method to gain advantage. We thoroughly evaluated the possibility of simultaneous excision of the main and metastatic lesions based on the findings of several preoperative exams. Additionally, the patient has gradually acquired tumor compression symptoms, including decreased appetite and post-eating abdominal distension. The patient’s quality of life would be poor and their estimated survival time would be short if surgery is not performed. Finally, we chose surgical treatment, and the postoperative pathological results showed that the surgery achieved R0 resection. At the same time, considering the tumor invading multiple organs with type II liver metastasis, postoperative adjuvant therapy was a more reliable treatment option. Based on the results of genetic testing and Chinese guidelines, we attempted the CAPTEM (capecitabine and temozolomide) chemotherapy. It is heartening that the surgical outcomes and the patient’s benefits match our expectations. The last issue that needs our attention is whether neoadjuvant treatment or transformation therapy may improve R0 resect pNETs with high tumor burden and high-risk recurrence and metastatic factors, benefiting patients’ survival rates. This may be a very fortunate case where our radical surgical perspective has not been validated through large-scale randomized controlled trials.

## Conclusion

We reported a G3 pNET with a high degree of biological malignancy, involving invasion of five adjacent organs, including the stomach, spleen, colon, adrenal gland, and kidney, as well as liver metastases. The surgeon should be inspired by this case report to distinguish between G3 pNETs and pNECs as well as to gain a fresh perspective on surgical indications. Through more proactive surgical procedures, providing survival benefits to patients with well-differentiated pNETs.

## Data availability statement

The original contributions presented in the study are included in the article/supplementary material. Further inquiries can be directed to the corresponding authors.

## Ethics statement

Written informed consent was obtained from the patient/participant for the publication of this Case Report.

## Author contributions

CZ, WN, YX contributed equally to the conception and design of the study and wrote the manuscript as co-first authors. YL, LH and SL cared for the patient. JW and XJ performed the surgical resection and reviewed the manuscript. All authors contributed to the article and approved the submitted version.
